# The Revised Identity Style Inventory: Factor Structure and Validity in Italian Speaking Students

**DOI:** 10.3389/fpsyg.2016.00883

**Published:** 2016-06-15

**Authors:** Lucia Monacis, Valeria de Palo, Maria Sinatra, Michael D. Berzonsky

**Affiliations:** ^1^Department of Human Sciences, University of Foggia, FoggiaItaly; ^2^Department of Educational Sciences, Psychology, Communication, University of Bari Aldo Moro, BariItaly; ^3^Department of Psychology, State University of New York at Cortland, Cortland, NYUSA

**Keywords:** identity styles, factorial structure, convergent validity, discriminant validity, psychometric properties

## Abstract

The purpose of this study was to evaluate the factor structure and convergent and discriminant validity of scores on an Italian translation of the Revised Identity Style Inventory (ISI-5) with samples of 237 adolescents (50 males, *M*_age_ = 18.04, *SD* = .86) and 268 university students (42 males, *M*_age_ = 22.71, *SD* = 3.70). Confirmatory Factor Analysis indicated that a three-factor solution provided a good fit, which was invariant across age and sex groups. The theoretically relationships between scores on the ISI and scores on measures of reasoning and identity processes, identity commitment, and social desirability were partially consistent, thus further studies are needed to give more evidence to the convergent and discriminant validity.

## Introduction

According to Erikson (1968), lifespan model of psychosocial development forming a coherent and stable sense of identity is the major developmental task that adolescents face. Berzonsky’s (1988, 1990) social-cognitive model postulates that there are stylistic differences in how adolescents approach or attempt to evade the task of forming, maintaining, and/or revising their sense of identity. Some youth have an *Informational Identity Style* and actively seek out and explore identity alternatives and deliberately process and evaluate identity-relevant information before personally making identity commitments about who they are and what they value and believe. Others have a *Normative Identity Style* and form identity commitments by more automatically internalizing and adhering to the values, standards, and expectations held for them by significant others, especially parents. Youth with a *Diffuse–avoidant Identity Style* procrastinate and attempt to delay dealing with identity conflicts and problems as long as possible. When they have to act and make choices their behavior is influenced mainly by situational demands and consequences rather than informed reasons or normative standards (Berzonsky and Ferrari, 2009). A voluminous body of research has been conducted on identity styles over the past 25 years (see Berzonsky, 2011 for a review). Research reveals that the informational style is associated with experiential openness, rational processing, cognitive complexity, self-chosen commitments, problem-focused coping strategies, vigilant decisional strategies, and self-transcendent values (Berzonsky, 2004, 2011; Berzonsky and Papini, 2014). The normative style is associated with firm goals and commitments, conscientiousness, self-control, conventional and traditional values, and a low tolerance for ambiguity and a strong desire for structure (Berzonsky, 2004, 2011; Soenens et al., 2005). A diffuse–avoidant style is related to weak commitments and goals, self-handicapping behaviors, hedonistic values, and an external locus of control (Berzonsky and Ferrari, 1996, 2009).

Identity styles are operationalized with a self-report Identity Style Inventory (ISI). Although various revisions and translated versions of the ISI have been used in more than twenty countries (Berzonsky, 2011), the internal reliabilities of the translated versions of the scales, especially the normative scale, have been less than .60 in some cases. In addition, because some of the scale statements focus on particular content areas such as political or religious beliefs, some items may be more relevant to some participants than others. To address these potential limitations Berzonsky et al. (2013) developed and validated a new version, the Revised Identity Style Inventory (ISI-5), which includes statements about generic identity categories (e.g., values, goals, and beliefs) rather than specific identity domains (e.g., religion, political beliefs, occupation, etc.), which should provide a more cultural-free assessment of identity styles. Scores on the English version of ISI-5 has been found to have excellent psychometric properties (Berzonsky et al., 2013).

Only a few studies have been conducted on identity style in Italian contexts (e.g., Crocetti et al., 2009, 2012, 2013; Monacis et al., 2012). This dearth of empirical research may be due to how few psychometrically sound Italian instruments for assessing the process of identity construction are available and the psychometric limitations linked with the domain-specificity of some items in the Italian version of the ISI-3.

The present investigation was designed to translate the fifth version of the Inventory (ISI-5) into Italian and examine its psychometric properties with Italian samples of adolescents and university students. More specifically, we sought to evaluate the factorial structure of the Italian version of ISI-5 and determine whether it was invariant across age and sex groups. Further, we examined the associations between the translated style scales and measures of identity and cognitive-reasoning processes and social desirability to evaluate the convergent and discriminant validity of scores on the translated style scale. We included measures of rational reasoning, automatic reasoning, identity commitment, and identity exploration as convergent-validity criteria and social desirability to assess discriminant validity. Based on previous validation research on identity styles (Berzonsky’s, 1990; Berzonsky, 2011; Crocetti et al., 2009; Phillips, 2009; Berzonsky et al., 2013), we predicted the following: (i) informational style scores would be associated positively with rational and experiential reasoning, identity exploration and commitment and negatively (or unrelated) to social desirability; (ii) normative style scores would be correlated positively with identity commitment and experiential reasoning and negatively (or unrelated) with identity exploration, rational reasoning, and social desirability; and (iii) diffuse–avoidant style scores would be associated negatively with rational reasoning and identity commitment and exploration and unrelated with experiential reasoning and social desirability.

## Materials and Methods

### Participants and Procedure

The original sample comprised 545 Italian students recruited via convenience sampling. Forty participants were excluded because of missing data. The final sample included 92 males and 413 females (*M*_age_ = 20.52, *SD* = 3.61). There were 237 adolescents (50 males and 187 females) who varied in age from 16 to 19 years (*M*_age_ = 18.04, *SD* = 0.86), and 268 university students (42 males and 226 females) who varied in age from 20 to 25 years (*M*_age_ = 22.71, *SD* = 3.70). The adolescents attended classical, scientific, and technical high schools and the university students attended various faculties (Humanities, Educational, and Social Sciences).

The Statement of 2014, 10 December of the Ethics Committee of the Department of Human Sciences from University of Foggia approved this study. Permission was required from heads and deans to conduct the research study at the school/institution. Written informed consent was obtained from students over 18 years of age; parents or legal guardians provided written consent for students under 18 years of age to participate. Respondents were asked to complete an anonymous questionnaire during an ordinary 50-min classroom lesson (no identifiers were collected). Data collection took place over three-month period. Potential order effects were controlled by presenting the questionnaires in three randomized orders.

### Instruments

Participants completed a self-report questionnaire that included a socio-demographic section and a battery of measures designed to assess identity style, identity processes, cognitive reasoning, and social desirability. As recommended by Merenda (2006), scales were translated from English into Italian separately by the Italian authors of this study. After the battery of measures was translated into Italian, it was then back-translated into English by a native speaker of English to establish their comparability.

Identity styles were assessed with the Revised Identity Style Inventory (ISI-5) developed by Berzonsky et al. (2013). In this version, all items are worded in the present tense and refer to one’s current identity processing style. Participants responded to the items on a 1 (not at all like me) to 5 (very much like me) Likert scale. Sample items include: “I handle problems in my life by actively reflecting on them” for the 9-item informational scale (α = 0.73); “I think it is better to adopt a firm set of beliefs than to be open-minded” for the 9-item normative scale (α = 0.66); and “Who I am changes from situation to situation” for the 9-item diffuse–avoidant scale (α = 0.72). The Inventory also includes a 9-item identity commitment scale (α = 0.79); (e.g., “I know basically what I believe and don’t believe”). Confirmatory Factor Analysis (CFA) indicated that a single-factor solution provided an adequate fit to the data, χ^2^ (2) = 10.61, *p* < 0.01; CFI = 0.99; SRMR = 0.02; RMSEA = 0.08, 90% CI [0.043, 0.092]. Factor loadings ranged from 0.66 to 0.82. Reliability and validity data relevant to scores on the four ISI-5 scales are presented in Berzonsky et al. (2013).

Rational and automatic reasoning processes were assessed with the Rational/Experiential Multimodal Inventory developed by Norris and Epstein (2011). It consists of 42 items rated on a 1 (completely false) to 5 (completely true) point Likert scale. It consists of a 12-item rational reasoning scale (α = 0.77; e.g., “I enjoy intellectual challenges”) and a 30-item experiential scale (α = 0.78; e.g., “I like to rely on my intuitive impressions”). This experiential scale is a revised and broadened version of the measure of intuitive-experiential reasoning developed by Epstein et al. (1996), which is associated with automatic reasoning processes such as superstitious reasoning and stereotyping. CFA indicated that the fit indices of the two-factor model were adequate, χ2 (9) = 45.54, *p* < 0.001; CFI = 0.93; SRMR = 0.07; RMSEA = 0.09, 90% CI [0.065, 0.116].

Identity exploration and commitment were assessed with an Italian translation of the Ego Identity Process Questionnaire (EIPQ: Balistreri et al., 1995). The EIPQ includes a 16-item identity exploration scale (e.g., “I have consistently re-examined many different values in order to find the ones which are best for me”) and a 16-item identity commitment scale (e.g., “I have definitely decided on the occupation I want to purse”) that are rated on a 1 (strongly disagree) to 6 (strongly agree) point Likert scale. A CFA on an Italian translation of the 32 items did not support a two-factor solution, χ^2^ (19) = 286.78, *p* < 0.001; CFI = 0.62; SRMR = 0.11; RMSEA = 0.16, 90% CI [0.150, 0.185]. The sample was randomly divided into two subsamples and a Principal Components Analysis was performed on all the items with the first subsample. Eighteen items with low loadings (<0.30) or cross-loadings were excluded and a CFA was performed on the remaining 14 items (7 exploration and 7 commitment items) with the second subsample of participants. The results indicated that the two-factor solution was adequate, χ^2^ (74) = 152.39, *p <* 0.001; CFI = 0.91; SRMR = 0.08; RMSEA = 0.05, 90% CI [0.050, 0.079]. Cronbach’s α was 0.60 for Exploration and 0.62 for Commitment.

The Italian version of the *Marlow–Crowne Social Desirability Scale* (MC-SDS; Crowne and Marlow, 1960; Manganelli Rattazzi et al., 2000) was used to assess social desirability. Participants responded “true” or “false” to 33 statements (e.g., “I never hesitate to go out of my way to help someone in trouble”, “It is sometimes hard for me to go on with my work if I am not encouraged”). Validity data for scores on the MC–SDS are reported by Manganelli Rattazzi et al. (2000). Internal reliability in the present study was 0.64.

## Results

### Preliminary Analyses

Scores on the diffuse–avoidant scale were negatively correlated with informational scores (*r* = -0.15, *p* < 0.001) and positively correlated with normative scores (*r* = 0.35, *p* < 0.001). Scores on the normative and informational scales were not correlated (*r* = 0.032, *p* = 0.446). Because some age and sex differences on identity style scores have been reported (Berzonsky, 2011), a Multivariate Analysis of Variance (MANOVA) was performed with the sex and age as independent factors and the three identity styles as the dependent variables. Only a main effect of age was found, Pillai’s Trace = 0.025, *F*(3, 499) = 4.221, *p* = 0.006, η^2^ = 0.025. Univariate analyses indicated that university students had higher informational scores (*M* = 35.88; *SD* = 4.59) than adolescents (*M* = 34.26; *SD* = 5.34), *F*(1, 504) = 7.33, *p* = 0.007, η^2^ = 0.014, but lower diffuse–avoidant scores (*M* = 18.59 *SD* = 6.27 versus *M* = 19.48; *SD* = 5.50, respectively), *F*(1, 504) = 3.90, *p* = 0.049, η^2^ = 0.008.

### Factor Structure of the Italian Version of the Inventory

To evaluate the three-factor structure of the 27 identity style items, a Confirmatory Factor Analysis (CFA) was performed using Amos (Arbuckle, 2006). Parcelling technique was applied since “models based on parcelled data (a) are more parsimonious (i.e., have fewer estimated parameters both locally in defining a construct and globally in representing an entire model), (b) have fewer chances for residuals to be correlated or dual loadings to emerge (both because fewer indicators are used and because unique variances are smaller), and (c) lead to reductions in various sources of sampling error” (Little et al., 2002, p. 155). Before creating parcels, saturation score for each item in each factor was checked. All factor loadings were significant at *p* < 0.001 and the average of the standardized factor loadings was 0.46 (**Table 1**).

**Table 1:** Standardized factor loadings of the ISI-5.

	Normative style	Informational style	Diffuse–avoidant style
ISI_2	0.203		
ISI_6	0.494		
ISI_10	0.180		
ISI_12	0.576		
ISI_16	0.427		
ISI_21	0.350		
ISI_25	0.464		
ISI_29	0.615		
ISI_31	0.361		
ISI_5		0.322	
ISI_9		0.475	
ISI_15		0.574	
ISI_20		0.592	
ISI_24		0.568	
ISI_28		0.594	
ISI_30		0.445	
ISI_34		0.541	
ISI_36		0.301	
ISI_35			0.600
ISI_33			0.507
ISI_32			0.510
ISI_26			0.626
ISI_22			0.611
ISI_17			0.468
ISI_13			0.401
ISI_7			0.284
ISI_3			0.279

Three parcels were then created for each latent variable, i.e., identity style, with a balancing approach in which the scale item with the highest item-scale correlation was combined with the two scale items with the lowest item-scale correlation (Little, 2013). Each parcel included three items. The CFA indicated that the three-factor solution provided a good fit to the data (**Table 2**). All factor loadings were significant and ranged from 0.58 to 0.80.

**Table 2:** Fit indices of the three-factor model for total sample, gender, and age groups.

Sample	*χ* ^2^	*df*	CFI	SRMR	RMSEA (90% C.I.)
Total sample (*N* = 505)	31.33 (*p* = 0.14)	24	0.99	0.03	0.02 (0.000–0.046)
Adolescents (*N* = 237)	12.13 (*p* = 0.98)	24	1.00	0.03	0.00 (0.000–0.000)
University students (*N* = 268)	61.79 (*p* < 0.000)	24	0.94	0.06	0.08 (0.053–0.101)
Males (*N* = 92)	31.12 (*p* = 0.15)	24	0.97	0.07	0.06 (0.000–0.108)
Females (*N* = 413)	34.31 (*p* = 0.08)	24	0.99	0.03	0.03 (0.000–0.055)

CFAs of the three-factor solution were performed separately for each age and sex subgroup. Results indicated that the three-factor model provided a good fit to the data for each subgroup (**Table 2**). To evaluate the generalizability of the three-factor model across male and female and adolescent and university student participants, two multi-group CFAs using maximum likelihood estimation were performed. For each analysis an unconstrained model, in which factor loadings were allowed to vary between each subgroup, was compared to a constrained model, in which the factor loadings were held constant for each subgroup. No significant differences were found indicating that the three-factor model fit equally well for male and female participants (Δχ^2^ = 8.25, Δd*f* = 6, *p* = 0.99; ΔCFI = 0.00) and adolescents and university students (Δχ^2^ = 4.62, Δd*f* = 6, *p* = 0.59; ΔCFI = 0.00).

### Convergent and Discriminant Validity

Once the three factor solution was confirmed, the extent to which the items of a specific factor converge or share a high proportion of variance was assessed through the Average Variance Extracted (AVE) method. In addition, the extent to which a factor was distinct from other factors both in terms of how much each factor correlated with other factors and how items represented distinctly only the single factor was evaluated through the comparison between the AVE and the Maximum Shared Squared Variance (MSV) of each factor (Farrell, 2010).

The AVE values were 0.51 for the Informational factor, 0.50 for the Diffuse–avoidant factor, and 0.44 for the Normative factor, thus indicating some limitations for the Normative dimension. Results from the comparison between AVE and MSV indicated that the MSV values of the three dimensions (Informational 0.042, Normative 0.20, and Diffuse–avoidant 0.042) were smaller than the AVE values.

To evaluate the convergent validity of scores on the style scales, correlations were computed between scores on the style variables and on the reasoning and identity measures (**Table 3**). As predicted, an informational style was positively related to identity exploration, rational and experiential reasoning, and identity commitment as measured by the ISI-5 scale. The normative style, as hypothesized, was positively associated with EIPQ identity commitment and negatively correlated with rational reasoning, especially among university students. Diffuse–avoidance, as expected, was negatively associated with identity exploration, rational reasoning, both EIPQ and ISI-5 commitment. Contrary to prediction, normative scores were not associated with ISI-5 commitment and experiential reasoning.

**Table 3:** Bivariate correlations between ISI-5, EIPQ, SD and ISI-Commitment.

Validity measures	Informational			Normative			Diffuse–avoidant		
	Total sample	Adol.	U.S.	Total sample	Adol.	U.S.	Total sample	Adol.	U.S.
**Ego Identity processes**
Exploration	0.27**	0.36**	0.18**	-0.07	0.06	-0.18**	-0.13**	-0.14*	-0.12*
Commitment	0.01	0.05	-0.04	0.25**	0.22**	0.27**	-0.10*	-0.02	-0.15*
**Cognitive reasoning processes**
Rational	0.32**	0.29**	0.33**	-0.12**	0.00	-0.23**	-0.31**	-0.28**	-0.33**
Experiential	0.23**	0.20**	0.21**	-0.06	-0.01	-0.10	-0.01	0.05	-0.04
**Social desirability**
Social desirability	-0.03	-0.13	0.09	0.05	0.06	0.05	0.11*	0.26**	-0.01
**Commitment (ISI-5)**
Commitment	0.24**	0.17**	0.27**	0.01	0.08	-0.05	-0.54**	-0.44**	-0.61**

Adol., Adolescents; U.S., University Students; **p < 0.01; *p < 0.05.

Because of covariance between the diffuse–avoidant and the normative styles (*r* = 0.35, *p* < 0.001), partial correlations with the ISI-5 commitment and experiential reasoning validation variables were computed for each style with the effects of the other style statistically controlled. The partial correlation between a normative style and ISI-5 commitment was significant for the entire sample (pr = 0.24, *p* < 0.001) as well as for adolescents (pr = 0.24, *p* < 0.001) and university students separately (pr = 0.25, *p* < 0.001) indicating that this relationship was suppressed by diffuse–avoidance. However, the partial relationship between the normative style and experiential reasoning remained non-significant (pr = -0.08, *p* = 0.077) providing no evidence that diffuse–avoidance suppressed the hypothesized relationship. The unexpected significant negative association between diffuse–avoidance and EPIQ exploration was reduced to non-significance when the effects of the informational and normative styles were statistically controlled (pr = -0.075, *p* = 0.093).

The pattern of correlations between social desirability and ISI styles generally supported the discriminant validity of scores on the style measures: social desirability was not significantly associated with scores on the normative or informational style scales. Although a significant relationship was obtained between scores on the diffuse–avoidant and social desirability scales, the effect size was extremely small (*r^2^* = 0.01, *p* = 0.010).

Taken together, the evidence supports the convergent and discriminant validity of the measurement model and the factor scores, with some limitations in the Normative factor.

## Discussion

In light of the increasing research interest in identity formation, the current study sought to assess the factor structure, internal reliability, and the convergent and discriminant validity of the scores of high school and university students on an Italian translation of the ISI-5. A CFA on the 27 translated style items indicated that the three-factor solution provided a good fit, which was consistent across male and female and high school and university students. The internal reliabilities of scores on the three style scales were acceptable, although the values were somewhat lower than those reported for scores on the American version of the ISI-5 (Berzonsky et al., 2013).

Convergent validity was assessed by determining whether scores on the translated style scales and measures of rational and experiential reasoning and identity processes were associated in a theoretically hypothesized manner. In general, the pattern of convergent-validity coefficients was consistent with expectations based on identity style theory (Berzonsky’s, 1990; Berzonsky, 2011). Informational scores were positively correlated with identity exploration, strength of identity commitment, and rational and experientially based automatic reasoning. Contrary to prediction, informational scores were not associated with EIPQ identity commitment. As hypothesized, normative scores were correlated positively with EIPQ commitment and negatively with rational reasoning. Contrary to prediction, normative scores were unrelated to ISI commitment and experiential reasoning. Diffuse–avoidant scores, as expected, were negatively correlated with identity exploration and commitment and rational reasoning and unrelated with experiential reasoning. Although, contrary to prediction, diffuse–avoidant scores were negatively associated with identity exploration; when the effects of normative and informational style scores were controlled the relationship was non-significant.

Because scores on the normative and diffuse–avoidant scales were positively correlated, ancillary analyses were conducted to further examine some of the anomalous findings. These analyses revealed that diffuse–avoidant scores suppressed the positive relationship between normative scores and ISI commitment. However, the partial relationship between normative scores and experiential reasoning, controlling for diffuse–avoidance, remained non-significant. This latter finding is inconsistent with the results of numerous studies (see Berzonsky, 2011; Berzonsky et al., 2013 for reviews). One explanation of this anomalous finding is that the revised version of experiential reasoning used in the present study (REIm: Norris and Epstein, 2011) was intentionally designed to assess adaptive aspects of experiential reasoning rather than the more rigid and inflexible superstitious and stereotypic reasoning associated with scores on the previous version developed by Epstein et al. (1996), which has been consistently found to correlate positively with the normative style. As predicted by identity style theory (Berzonsky, 2011), the present results indicate that the normative style is not associated with the more flexible, adaptive experiential reasoning purportedly measured by the REIm. It would be useful in future research to investigate whether scores on the Italian normative scale are positively associated with measures of more closed, inflexible automatic reasoning such as other measures of intuitive reasoning (e.g., Epstein et al., 1996) and Need for Cognitive Closure (Webster and Kruglanski, 1994; Soenens et al., 2005).

The discriminant validity of scores on the style scales was evaluated by examining correlations with social desirability. Consistent with previous research (Phillips, 2009), neither informational nor normative scores were associated with social desirability. Although diffuse–avoidant scores were positively related with social desirability the effect size was extremely small.

Caution must be exercised in interpreting these results. Even if these findings supported the original structure of the Inventory, there are just a few items showing factor loadings below 0.30. Clearly, further studies should improve these problematic items and, thus, replicate the analyses with other samples. Although a multi-groups analysis indicated that the three-factor structure was invariant across male and female participants, the sample was heavily unbalanced in favor of females (82%). Accordingly, there is a need to replicate the findings with a much larger sample of male participants to evaluate the cross-gender generalizability of the findings. In addition, the samples were relatively homogeneous high school and university students. An important direction for future research is to continue to evaluate the psychometric properties of the ISI-5 scales across probability samples with more diverse ages and cultural, educational, and socio-economic backgrounds. Finally, the inconsistent associations between the constructs of interest may be due to the revision of the original EIPQ as described in the instruments section, and to the new measure of the experiential reasoning process, i.e., the REIm. Future research should attempt to replicate the current study using alternative measures of identity exploration and commitment and automatic reasoning.

In sum, these psychometric data show that the Italian version of the ISI-5 is a useful instrument for investigating identity processing styles. However, the findings indicate that investigators need to vigilantly examine and control the covariance between the style scales, especially the common variance shared by scores on the normative and diffuse–avoidant scales. Unlike the ISI-3, ISI-5 does not include items that deal with domain-specific content such as religion or occupation. Therefore, the more generic ISI-5 scales are likely to be more useful than the ISI-3 scales for longitudinal and cross-national research with participants from diverse background or of different ages. Indeed, the present findings indicate that the psychometric properties of the Italian ISI-5 were comparable for both teenage high school students and older university students. Finally, the findings provide additional evidence for the cross-national generalizability of the factor structure of the ISI-5 scales.

## Conclusion

The given multifaceted suitability of the ISI-5 it can be applied in different settings. For example, the ISI has been widely used to investigate relationships between identity styles and academic and social participation in educational contexts (Kaczan et al., 2013), recovery from substance abuse (White et al., 2003), and cultural transitions (Szabo and Ward, 2015). The ISI-5 provides Italian researchers with an objective means of investigating the role that individual differences in identity exploration and construction may play in predicting adaptive/maladaptive behaviors across a variety of contexts and situations.

## Author Contributions

LM: Substantial contributions to the conception and design of the work and acquisition of data; VdP: analysis, and interpretation of data; MS and MB: Drafting the work or revising it critically for important intellectual content and for final approval of the version to be published

## Conflict of Interest Statement

The authors declare that the research was conducted in the absence of any commercial or financial relationships that could be construed as a potential conflict of interest.
